# Nonerythropoietic Erythropoietin Mimetic Peptide ARA290 Ameliorates Chronic Stress-Induced Depression-Like Behavior and Inflammation in Mice

**DOI:** 10.3389/fphar.2022.896601

**Published:** 2022-08-15

**Authors:** Guanglei Xu, Tao Zou, Lijiao Deng, Guang Yang, Tingting Guo, Yi Wang, Chunxiao Niu, Qianqian Cheng, Xiqin Yang, Jie Dong, Jiyan Zhang

**Affiliations:** ^1^ Beijing Institute of Basic Medical Sciences, Beijing, China; ^2^ Chinese Institute for Brain Research, Beijing, China

**Keywords:** depression, chronic stress, EPO, ARA290, inflammation

## Abstract

Major depressive disorder (MDD) is a highly prevalent psychiatric disorder. But the treatment of depression remains challenging. Anti-inflammatory treatments frequently produce antidepressant effects. EPO-derived helix-B peptide ARA290 has been reported to retain the anti-inflammatory and tissue-protective functions of EPO without erythropoiesis-stimulating effects. The effects of ARA290 on MDD remain elusive. This study established chronic unpredictable mild stress and chronic social defeat stress mouse models. Daily administration of ARA290 during chronic stress induction in two mouse models ameliorated depression-like behavior, similar to fluoxetine. With marginal effects on peripheral blood hemoglobin and red cells, ARA290 and fluoxetine reversed chronic stress-induced increased frequencies and/or numbers of CD11b^+^Ly6G^hi^ neutrophils and CD11b^+^Ly6C^hi^ monocytes in the bone marrow and meninges. Furthermore, both drugs reversed chronic stress-induced microglia activation. Thus, ARA290 ameliorated chronic stress-induced depression-like behavior in mice through, at least partially, its anti-inflammatory effects.

## Introduction

Major depressive disorder (MDD) is a highly prevalent psychiatric disorder. But the treatment of MDD remains challenging (Chisholm et al., 2004; Smith, 2014). Stressful life events, especially those involving early-life abuse and social rejection, are one of the strongest proximal risk factors for MDD (Kendler et al., 2003). Numerous studies have demonstrated that psychosocial stress upregulates components of the immune system involved in inflammation (Syed et al., 2018; Beurel et al., 2020; Drevets et al., 2022). Excess or prolonged inflammation perturbs the synthesis, release, and reuptake of several neurotransmitters including serotonin (5-HT) (Lucki, 1998; Cipriani et al., 2009; Alboni et al., 2017). Although mostly distributed peripherally, a small proportion of 5-HT is present in the central nervous system and produces a general modulation of behavior across species (Lucki, 1998; Cipriani et al., 2009; Alboni et al., 2017). Selective serotonin reuptake inhibitors such as fluoxetine and paroxetine are the most commonly prescribed drugs for treating depression (Lucki, 1998; Cipriani et al., 2009; Alboni et al., 2017).

Erythropoietin (EPO) is a hypoxia-inducible growth factor predominantly produced by the kidney in adults (Broxmeyer, 2013). It is the main peptide that promotes erythropoiesis through the homodimer EPO receptor (EPOR_2_) (Krantz, 1991). EPO has increasingly been recognized to perform nonhematopoietic functions, including anti-inflammatory activities (Chen et al., 2014; Luo et al., 2016), promotion of neurodifferentiation and neuroplasticity (Wakhloo et al., 2020; Fernandez Garcia-Agudo et al., 2021), and vascular adaptation to pregnancy (Wolfson et al., 2019). It has been demonstrated that the anti-inflammatory and cytoprotective effects of EPO are realized through the heterodimeric EPOR/CD131 complex (Brines and Cerami, 2012; Bohr et al., 2013). Based on this mechanism, derivatives of erythropoietin that are tissue protective but not erythropoietic have been developed (Leist et al., 2004). For example, a peptide derived from the tertiary structure of EPO, ARA290 (sequence: QEQLERALNSS), was reported to retain the cytoprotective properties but lack the hematopoietic activity (Collino et al., 2015). The glutamine residue in the N-terminal position of ARA290 spontaneously undergoes cyclization into pyroglutamate. Despite a short plasma half-life (∼2 min) (Collino et al., 2015), ARA290 has been reported to show protective effects against several clinical diseases, such as type 2 diabetes (McVicar et al., 2011; Dahan et al., 2016; Muller et al., 2016), autoimmunity (Chen et al., 2014; Liu et al., 2014; Huang et al., 2018), transplantation rejection (Watanabe et al., 2016; Yan et al., 2018), and renal ischemia-reperfusion (van Rijt et al., 2013).

In the case of neuropathy, ARA290 has been demonstrated to attenuate small nerve fiber loss and neuritis caused by type 2 diabetes (McVicar et al., 2011; Dahan et al., 2013; Dahan et al., 2016), autoimmunity (Liu et al., 2014; Huang et al., 2018), or localized peripheral nerve inflammation (Pulman et al., 2013). In the central nervous system, intraperitoneal injection of 30 μg/kg of ARA290 once a day on days 1, 3, 6, 8, and 10 after spared nerve injury was reported to suppress the spinal microglia response and thereby produce long-term relief of neuropathic pain (Swartjes et al., 2014). Intraperitoneal injection of 0.7 nmol/kg ARA290 once a week for 5 weeks decelerates Alzheimer’s disease-like pathology progression with more monocyte progenitors in the bone marrow and the accumulation of Ly6C^low^ patrolling monocyte subset in the brain, which are implicated in clearing Aβ from the cerebral vasculature (Al-Onaizi et al., 2022). Intraperitoneal injection of 35 μg/kg of ARA290 once a day from day 7 to day 18 of experimental autoimmune encephalomyelitis induction could alter T-cell function to ameliorate clinical scores and tissue inflammation (Chen et al., 2014). Thus, systemic administration of ARAR290 can alleviate injuries in the central nervous system.

EPO administrated systemically can cross the blood–brain barrier (Brines et al., 2000) and produce antidepressant effects in rodents (Girgenti et al., 2009; Leconte et al., 2011). Furthermore, “depressive syndrome severity” in treatment-resistant depressed unipolar patients was ameliorated by eight weekly EPO infusions (*n* = 14) vs. saline groups (*n* = 17) (Miskowiak et al., 2014). Based on these findings, a previous study has investigated the possible antidepressant effects of ARA290 in a neuropsychological model of drug action (Cerit et al., 2015). Healthy volunteers (*n* = 36) received ARA290 (2 mg) or placebo in a double-blind, randomized, parallel-group design. No effects were observed on mood and affective symptoms one week later although ARA290 increased attention toward positive emotional pictures (Cerit et al., 2015). However, one single dose of ARA290 and the small number of cases limited the significance of the study. It is still of importance to clarify the possible antidepressant effects of ARA290.

In this study, we established chronic unpredictable mild stress (CUMS) and chronic social defeat stress (CSDS) depression-like mouse models. Here, we report that daily administration of 0.5 mg/kg of ARA290, a high dose previously used to ameliorate systemic lupus erythematosus induction in mice (Huang et al., 2018), during chronic stress induction in two mouse models ameliorated depression-like behavior with reduced inflammation.

## Materials and Methods

### Mice

All mice used in this study were bought from SPF (Beijing, China) Biotechnology Co., Ltd. All mice were maintained under specific pathogen-free conditions with controlled temperature (24 ± 1°C) and humidity (50 ± 10%) and a 12-h light–dark cycle. Mice were acclimatized through 7 days of adaptive feeding and then randomly assigned 4 groups, namely, control, model + PBS, model + fluoxetine, and model + ARA290 groups. Mice in model + fluoxetine, model + ARA290, and model + PBS groups were intraperitoneally injected once a day with 10 mg/kg of fluoxetine (Cat# 064-04323, Wako Chemical Co. Ltd., Tokyo, Japan) dissolved in PBS, 0.5 mg/kg of ARA290 (Peptide Biochemical GmbH, Hangzhou, China; endotoxin-free with N90% purity) dissolved in PBS, or the same volume of PBS, respectively, during chronic stress induction. The contents of this study and the use of animals were approved by the ethics committee of the Beijing Institute of Basic Medical Sciences under the number AMMS 2021-1356.

### Chronic Unpredictable Mild Stress Model

For this model, 8-week-old male C57BL/6 mice were used. Mice other than those in the control group were subjected to the following 7 stress stimuli for 6 weeks: food deprivation for 24 h, water deprivation for 24 h, overnight illumination for 8 h, restraint stress for 5 h, cage tilting at 45° for 24 h, horizontal oscillation for 20 min, and a soiled cage environment (500 ml of water into 250 g of sawdust bedding). In particular, unpredictable pressure stimulation was used to make sure that mice were exposed to these stresses in random order each week.

### Chronic Social Defeat Stress Model

Retired breeders of male CD-1 mice were individually housed and were selected for modeling if they attack C57BL/6 mice with latency ≤30 s for at least 2 consecutive days in 3 consecutive days. Special cages with perforated Plexiglas plates separating the space into two halves were used. An intruder 5-week-old male C57BL/6 mouse was directly exposed to a resident CD-1 mouse for 10 min. If the CD-1 mouse kept continuous biting even after the intruder mouse displayed submissive posturing, the defeat bout was immediately terminated. At the end of the frustration, the defeated C57BL/6 mouse was transferred to the other side of the cage so that the two mice could still maintain sensory and olfactory contact (Iñiguez et al., 2014). This procedure was repeated for 10 consecutive days with individual C57BL/6 mouse exposed to different CD-1 mice each day. C57 BL/6 mice in the control group were handled daily and housed in the same type of cages.

### Sucrose Preference Test

In this experiment, each mouse was individually housed with free access to food and water. During the first 48 h, the C57 BL/6 mice were habituated to drink from two bottles, one filled with 1% sucrose solution and the other containing tap water. On the third day, water and sucrose consumption was measured. The positions of the sucrose solution and the tap water were exchanged every 12 h and counterbalanced across the different cages. The consumption of sucrose solution and tap water was recorded respectively and the sucrose preference was calculated using the following equation: total liquid intake (g) = sucrose solution intake (g) + tap water intake (g); preference = [sucrose solution intake (g)/total liquid intake (g)] × 100% (Sun et al., 2019).

### Forced Swim Test

A forced swim test was performed as previously described (Can et al., 2012). In brief, each C57 BL/6 mouse was put into a cylindrical glass tank with a height of 50 cm and a diameter of 20 cm. The cylindrical tank was cleaned before each mouse was tested. Then it was filled with tap water which was kept at 23–25°C. The water level is 40 cm above the bottom to ensure that the mouse’s tail or feet cannot touch the bottom of the tank. Each mouse was put in the tank for 6 min. The criterion for immobility is that the mouse stops climbing/swimming in the water and does nothing but balance its body by the necessary movements to keep its nose out of the water. The accumulated immobility time of the mouse in the last 4 min was recorded.

### Tail Suspension Test

Mice were suspended 50 cm above the floor for 6 min with the tip (1 cm) of the tail attached using adhesive tape. The accumulated immobility time of the mouse in the last 4 min was recorded (Sun et al., 2019).

### Intravascular Immune Labeling

To label intravascular leukocytes, each mouse was intravenously injected with 2 μg of FITC-conjugated antimouse CD45 antibody (Cat# 103108, clone 30-F11, BioLegend, San Diego, CA, United States) via the tail vein. Mice were then anesthetized with 1% pentobarbital (80 mg per kilogram, Cat# P-010, Sigma-Aldrich, St. Louis, MO, United States) via intraperitoneal injection, followed by intracardiac perfusion 5 min later (Cugurra et al., 2021).

### Single-Cell Isolations

Mice were lethally sedated and perfused with ice-cold PBS for 5 min. Peripheral blood was collected before perfusion from the retroorbital sinus. After perfusion, the skull cap was quickly removed and the cranial dura was carefully peeled off with fine forceps. Small pieces of the cranial dura were quickly squeezed using tweezers (Cat# XYN-ZC/100, 66vision, Suzhou, China) in ice-cold RPMI-1640 containing 10% fetal bovine serum. The femur BM cells were flushed out of the bone marrow cavity using a 5 ml syringe with ice-cold PBS. Cells from these tissues were filtered through a 70-μm cell strainer. After centrifugation at 420*g* for 5 min, red blood cells were depleted by hypotonic lysis.

### Flow Cytometry Analysis

Single-cell suspensions were washed once with FACS washing buffer (2% FBS and 0.1% NaN_3_ in PBS). Cells were then incubated with fluorescence-conjugated antibodies against cell surface molecules for 30 min on ice. PERCP-CY5.5 antimouse CD45 (Cat# 103131, clone 30-F11), PE antimouse Ter119 (Cat# 116207, clone TER-119), Brilliant Violet 510 antimouse CD11b (Cat#101263, clone M1/70), Brilliant Violet 421 antimouse CD11c (Cat#117330, clone N418), APC-Cyanine7 antimouse Ly6G (Cat# 108424, clone RB6-8C5), and Brilliant Violet 605 antimouse Ly6C (Cat# 108440, clone HK1.4) were purchased from BioLegend. To determine cell viability, a LIVE/DEAD^™^ Fixable Green Dead Cell Stain Kit (Cat# L34970, Thermo Fisher Scientific, Waltham, MA, United States) was used according to the manufacturer’s instructions. After washing with FACS buffer, the cells were fixed with 1% (w/v) paraformaldehyde in PBS and preserved at 4 °C. Flow cytometry was performed using a Becton Dickinson FACS Fortessa machine (East Rutherford, NJ, United States) and the data were analyzed using the FlowJo version 10.

### Indirect Immunofluorescence

Mice were anesthetized and the brains were perfused with precooled saline and 4% (w/v) paraformaldehyde. Brains were then harvested and fixed for an additional 24 h with 4% (w/v) paraformaldehyde. Tissues were dehydrated with 15 and 30% sucrose solution and then embedded in OCT (Cat# 23-730-571, Thermo Fisher Scientific) and stored under refrigeration at −80°C. Then, 10 μM thick maximal prefrontal cortex coronal sections were sliced. For immunofluorescence staining, the largest tissue sections were retrieved from the refrigerator and rewarmed for 30 min in PBS. The nonspecific sites were blocked by incubation with 5% BSA and 0.3% Triton X-100 in PBS for 1 h at room temperature. Samples were then incubated with an antibody against ionized calcium-binding adaptor molecule 1 (Iba1, 1:200 diluted in blocking buffer, Cat#019-19741, Wako Chemicals, Richmond VA, United States) overnight at 4°C. After being washed three times in PBS, the samples were incubated with a TRITC-conjugated secondary antibody for 45 min at room temperature. Samples were washed again as stated above, mounted with DAPI (ZLI-9557, Origene, Rockville, MD, United States), and then observed under a laser scanning confocal microscope (RADIANCE 2100, Bio-Rad, Hercules, CA, United States).

### Quantitative RT-PCR

Total RNA was extracted with Trizol reagent (Cat# 15596026, Life Technologies, Carlsbad, CA, United States) and quantified using a Qubit RNA Assay Kit (Cat# Q32852, Life Technologies) in Qubit 2.0 Fluorometer. First-strand synthesis was performed with Oligo dT primers (Cat# 79237, Qiagen, Hilden, Germany) and reverse transcription was performed with M-MLV reverse transcriptase (Cat# 28025013, Thermo Fisher Scientific). Quantitative PCR was performed with SYBR Green reagent (Cat# QPK-101, TOYOBO, Tokyo, Japan) in a real-time PCR machine Realplex 2. The relative expression of the target genes was normalized to the *Gapdh* internal control (the 2^−ΔΔCt^ method). The following primers were used: murine *Tnfa*, 5′-GTG​GAA​CTG​GCA​GAA​GAG-3′ (forward) and 5′-GCT​ACA​GGC​TTG​TCA​CTC-3′ (reverse); murine *Il6*, 5′-GAT​GGA​TGC​TAC​CAA​ACT​GGA-3′ (forward) and 5′-TCT​GAA​GGA​CTC​TGG​CTT​TG-3′ (reverse); murine *Il10*, 5′-TTAATAAGCTCC AAGACCAAGG-3′ (forward) and 5′-CAT​CAT​GTA​TGC​TTC​TAT​GCA​G-3′ (reverse); and murine *Gapdh*, 5′-CCA​TCA​CCA​TCT​TCC​AGG​AGC​GAG-3′ (forward) and 5′-GAT​GGC​ATG​GAC​TGT​GGT​CAT​GAG-3′ (reverse).

### Statistical Analysis

Quantitative data are shown as mean ± standard deviations and were analyzed using Prism 6.0 (GraphPad). One-way ANOVA with posthoc analysis (Dunnett—compared to the ‘CUMS/CSDS + PBS’ group) was used to evaluate the quantitative variables. *p* < 0.05 was considered significant.

## Results

### ARA290 Reduced Chronic Unpredictable Mild Stress- and Chronic Social Defeat Stress-Induced Depression-Like Behavior

**FIGURE 1:**
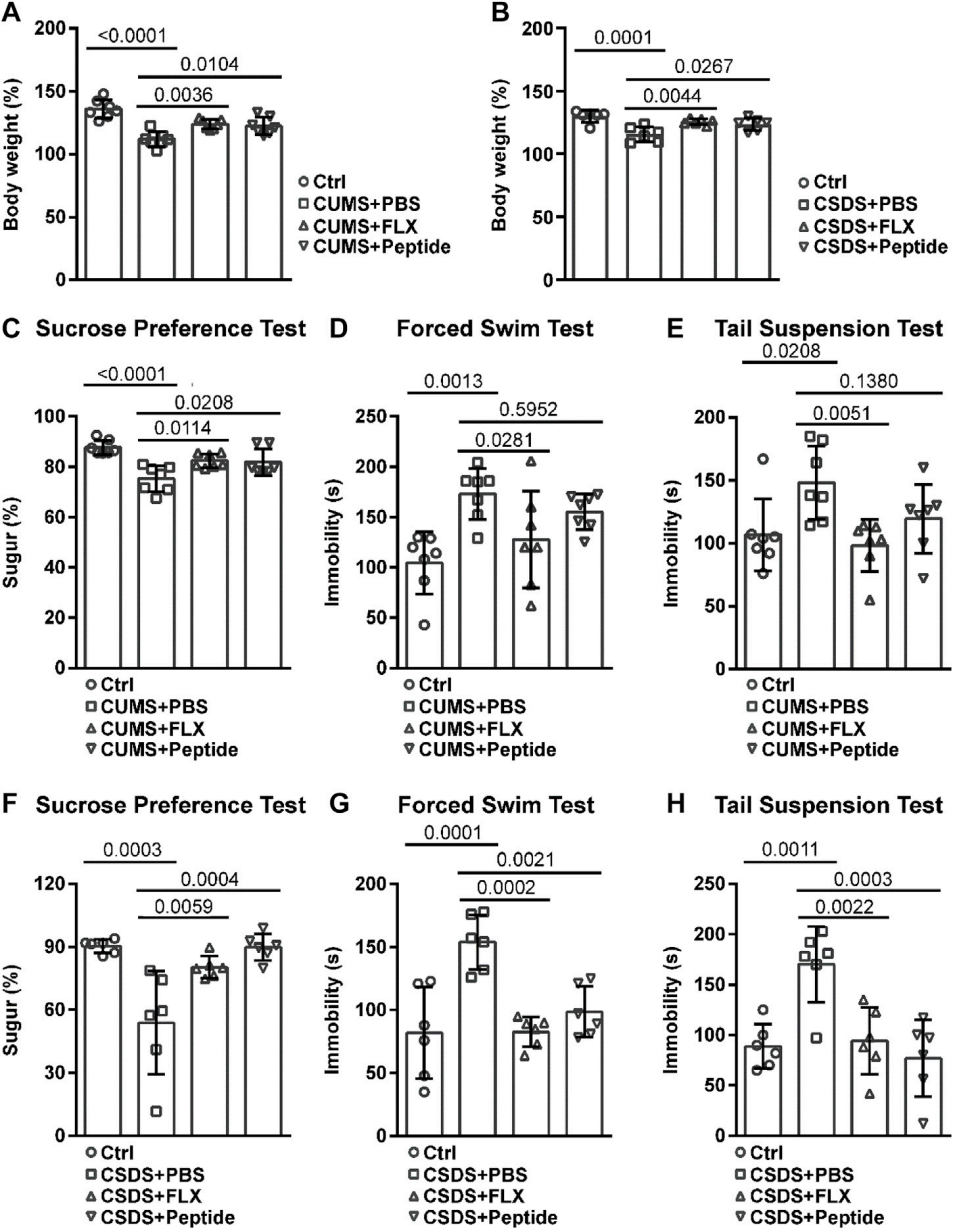
ARA290 reduced CUMS- and CSDS-induced depression-like behavior: 8-week-old male C57BL/6 mice subjected to the CUMS model (*n* = 7 mice per group) **(A and C–E)** or 5-week-old male C57BL/6 mice subjected to the CSDS model (*n* = 6 mice per group) **(B and F–H)** were intraperitoneally injected with fluoxetine (FLX, 10 mg/kg) or ARA290 (peptide, 0.5 mg/kg) once a day during chronic stress induction. Then, the body weight change **(A and B)**, sucrose preference rate **(C and F)**, and immobility time in the forced swim test **(D and G)** and the tail suspension test **(E and H)** were measured. The statistical values between different groups are shown.

To clarify the possible antidepressant effects of ARA290, we employed CUMS and CSDS depression-like mouse models. Both CUMS and CSDS led to reduced body weight, which was reversed by daily administration of fluoxetine or ARA290 during chronic stress induction (Figures 1A and B). Then we analyzed the depression-like behavior. As expected, both fluoxetine and ARA290 reversed CUMS-induced anhedonia, a core symptom of depression in humans, as indicated by diminished sucrose preference (Figure 1C). Fluoxetine also reversed CUMS-induced psychomotor retardation, as indicated by prolonged immobility in the forced swim test and the tail suspension test. ARA290 failed to affect CUMS-induced prolonged immobility in the forced swim test, but exhibited the tendency to reverse CUMS-induced prolonged immobility in the tail suspension test, although the effects failed to reach statistical significance (Figures 1D and E). As for the CSDS model, both fluoxetine and ARA290 reversed chronic stress-induced anhedonia (Figure 1F) and psychomotor retardation (Figures 1G and H).

### ARA290 Exhibited Marginal Effects on Peripheral Blood Hemoglobin and Red Cells in Chronic Unpredictable Mild Stress and Chronic Social Defeat Stress Mouse Models

**FIGURE 2:**
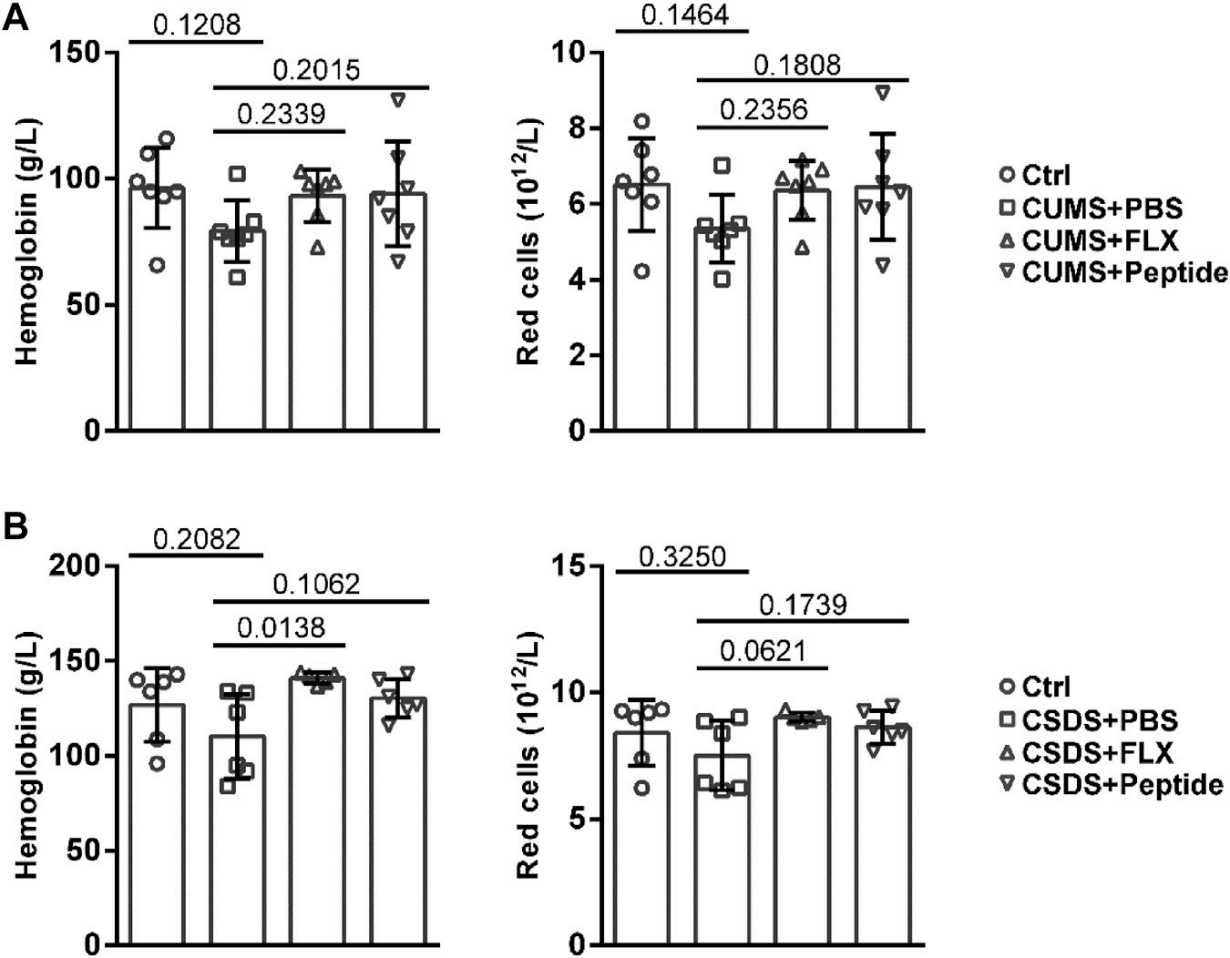
ARA290 exhibited marginal effects on peripheral blood hemoglobin and red cells in the CUMS and CSDS mouse models: 8-week-old male C57BL/6 mice subjected to the CUMS model (*n* = 7 mice per group) **(A)** or 5-week-old male C57BL/6 mice subjected to the CSDS model (*n* = 6 mice per group) **(B)** were treated with fluoxetine (FLX) or ARA290 (peptide) daily. The levels of hemoglobin and red cells in the peripheral blood were then measured. Please note that one sample in the CSDS + FLX group accidentally underwent coagulation. The statistical values between different groups are shown.

Then, we tested whether ARA290 stimulates erythropoiesis during chronic stress induction. Both CUMS and CSDS showed the tendency to reduce the levels of hemoglobin and red cells in the peripheral blood. Fluoxetine and ARA290 showed the tendency to increase the levels of peripheral blood hemoglobin and red cells under chronic stress (Figures 2A and B). However, statistical significance was reached or almost reached only for fluoxetine and only in the CSDS model. In other words, ARA290 exhibited marginal effects on peripheral blood hemoglobin and red cells in both CUMS and CSDS mouse models (Figures 2A and B).

### ARA290 Exhibited Similar Effects to Fluoxetine on Bone Marrow Erythropoiesis and Myelopoiesis in the Chronic Unpredictable Mild Stress Mouse Model

**FIGURE 3:**
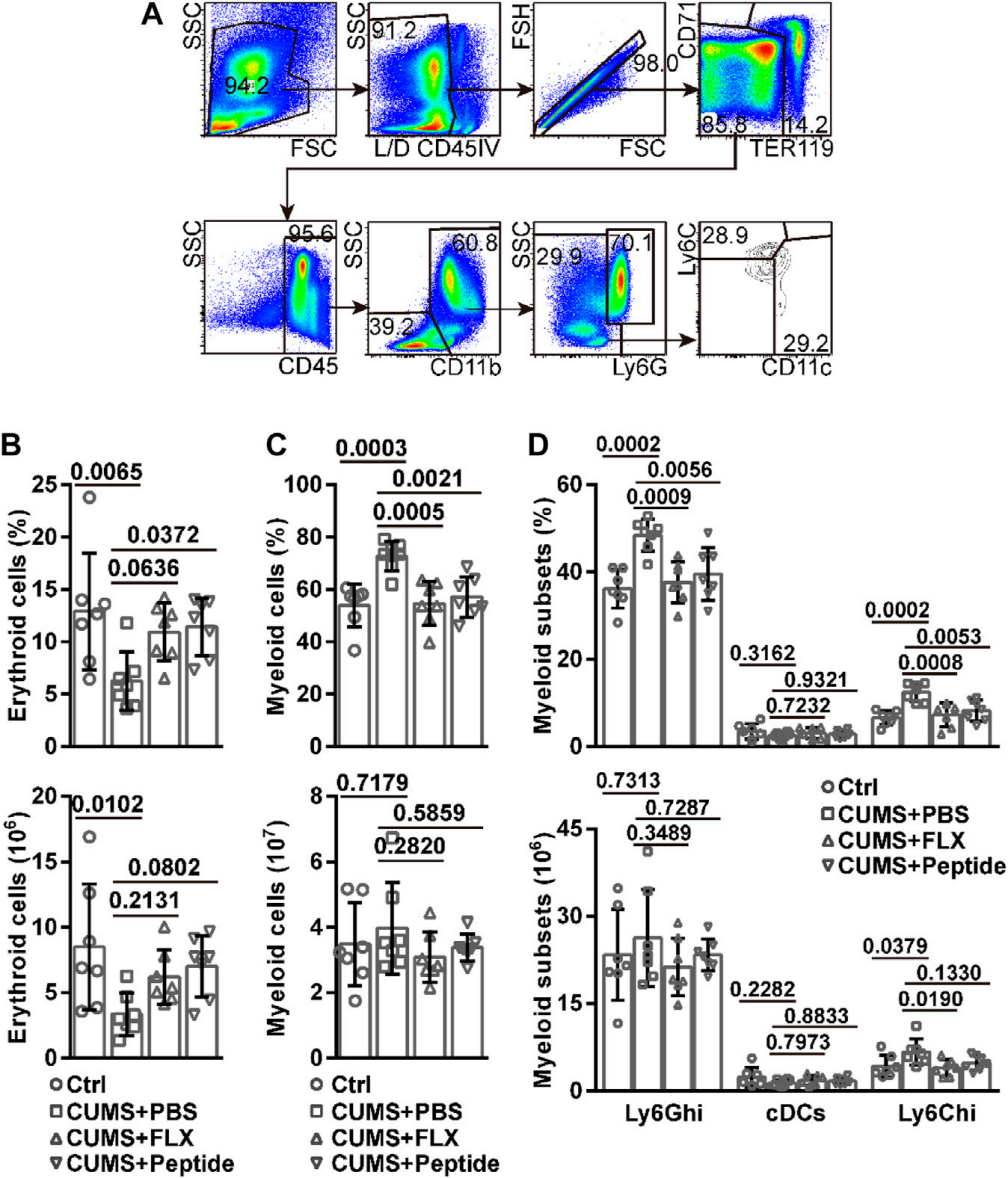
ARA290 exhibited similar effects to fluoxetine on bone marrow erythropoiesis and myelopoiesis in the CUMS mouse model: 8-week-old male C57BL/6 mice subjected to the CUMS model (*n* = 7 mice per group) were treated with fluoxetine (FLX) or ARA290 (peptide) daily. Then bone marrow cells were subjected to flow cytometry analysis of erythroid and myeloid cells. **(A)** representative gating strategy. **(B)** the frequency and number of Ter119^+^ or CD71^+^ erythroid cells. **(C)** the frequency and number of CD45^+^CD11b^+^ myeloid cells. **(D)** the frequency and number of CD11b^+^CD11c^−^Ly6G^hi^ neutrophils, CD11b^+^CD11c^−^Ly6C^hi^ monocytes, and CD11b^+^CD11c^+^ conventional dendritic cells (cDCs). The statistical values between different groups are shown.

Our aforementioned data promoted us to examine bone marrow erythropoiesis. Furthermore, several studies have reported chronic stress-induced neutrophilia and monocytosis (Powell et al., 2013; Heidt et al., 2014; Zhu et al., 2017). Therefore, we also investigated CD11b^+^CD11c^−^Ly6G^hi^ neutrophils, CD11b^+^CD11c^−^Ly6C^hi^ monocytes, and CD11b^+^CD11c^+^ conventional dendritic cells (cDCs) in the bone marrow (Figure 3A). The CUMS model resulted in a lower frequency and number of erythroid cells in the bone marrow. ARA290 reversed CUMS-induced lower frequency of erythroid cells in the bone marrow. Fluoxetine exhibited a similar tendency, although the effects failed to reach statistical significance. Both drugs also showed the tendency to reverse CUMS-induced lower number of erythroid cells in the bone marrow, although the effects failed to reach statistical significance (Figure 3B). Even though the CUMS model resulted in a higher frequency of myeloid cells in the bone marrow, the number of myeloid cells remained unchanged. Both fluoxetine and ARA290 reversed the higher frequency of myeloid cells without affecting their number in the bone marrow (Figure 3C). In this scenario, we analyzed individual myeloid subsets. The CUMS model resulted in a higher frequency of Ly6G^hi^ neutrophils and Ly6C^hi^ monocytes, but not cDCs, in the bone marrow. Both fluoxetine and ARA290 reversed CUMS-induced higher frequency of Ly6G^hi^ neutrophils and Ly6C^hi^ monocytes in the bone marrow and did not affect that of cDCs (Figure 3D). Regarding the number of these subsets, the CUMS model resulted in a higher number of Ly6C^hi^ monocytes, but not Ly6G^hi^ neutrophils and cDCs, in the bone marrow. Fluoxetine reversed CUMS-induced higher number of Ly6C^hi^ monocytes in the bone marrow. ARA290 exhibited a similar tendency, although the effects failed to reach statistical significance. Both drugs did not affect the number of Ly6G^hi^ neutrophils and cDCs in the bone marrow (Figure 3D).

### ARA290 Exhibited Similar Effects to Fluoxetine on Meningeal Myeloid Cells in the Chronic Unpredictable Mild Stress Mouse Model

**FIGURE 4:**
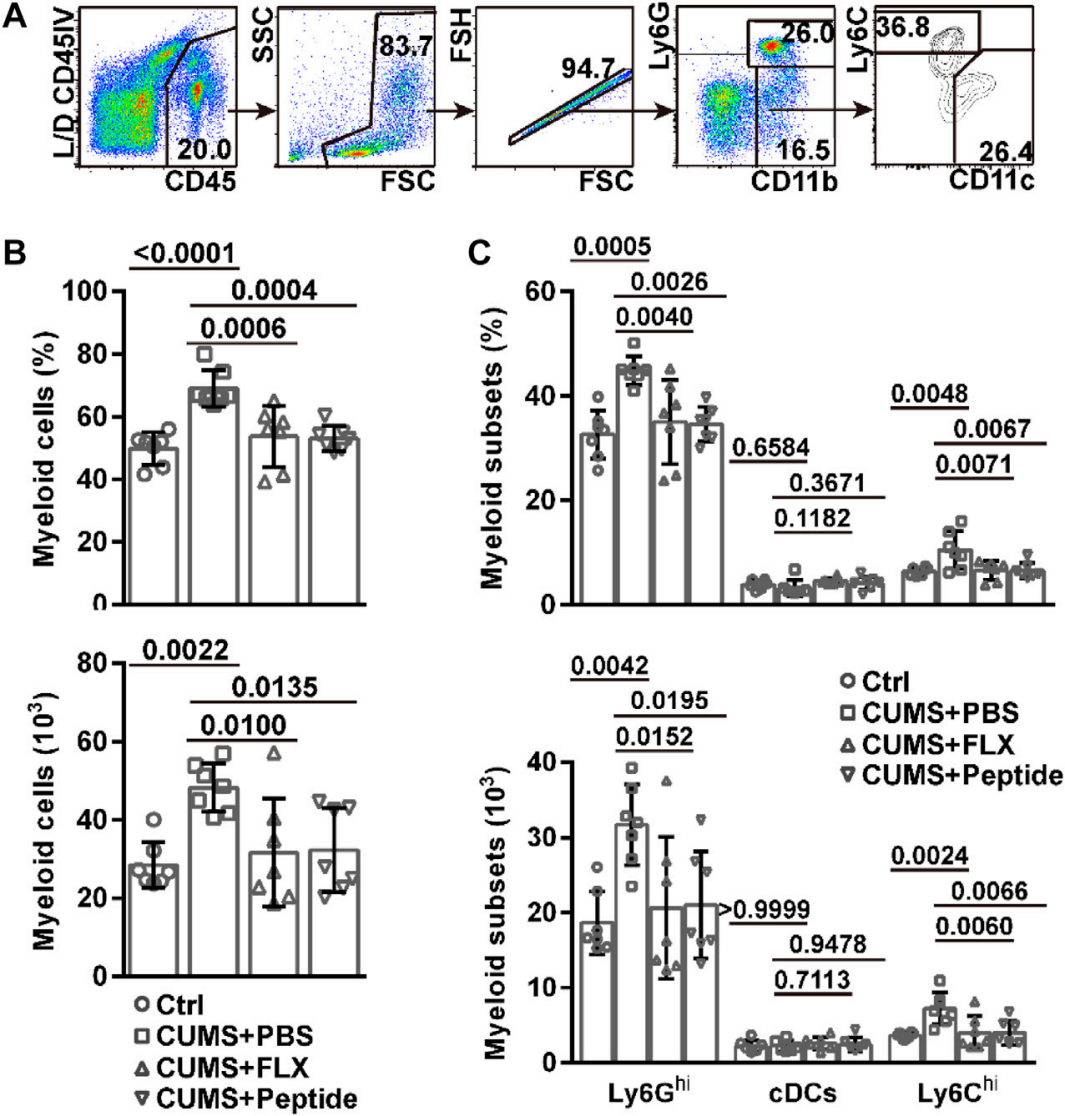
ARA290 exhibited similar effects to fluoxetine on meningeal myeloid cells in the CUMS mouse model: 8-week-old male C57BL/6 mice subjected to the CUMS model (*n* = 7 mice per group) were treated with fluoxetine (FLX) or ARA290 (peptide) daily. Then, mechanically isolated dura cells were subjected to flow cytometry analysis of myeloid cells. **(A)** representative gating strategy. **(B)** the frequency of CD11b^+^ myeloid cells in CD45^+^ leukocytes and their number in the cranial dura. **(C)** the frequency of CD11b^+^CD11c^−^Ly6G^hi^ neutrophils, CD11b^+^CD11c^−^Ly6C^hi^ monocytes, and CD11b^+^CD11c^+^ conventional dendritic cells (cDCs) in CD45^+^ leukocytes and their number in the cranial dura. The statistical values between different groups are shown.

In recent years, it has been disclosed that the meninges contain a pool of monocytes and neutrophils. Under the conditions of neuroinflammation, central nervous system-infiltrating myeloid cells can originate from brain borders (Cugurra et al., 2021). In this scenario, we also investigated CD11b^+^CD11c^−^Ly6G^hi^ neutrophils, CD11b^+^CD11c^−^Ly6C^hi^ monocytes, and CD11b^+^CD11c^+^ cDCs in the cranial dura (Figure 4A). The CUMS model resulted in a higher frequency and number of myeloid cells in the meninges. Both fluoxetine and ARA290 reversed the accumulation of myeloid cells in the meninges (Figure 4B). In detail, the CUMS model resulted in a higher frequency and number of Ly6G^hi^ neutrophils and Ly6C^hi^ monocytes, but not cDCs, in the meninges. Both fluoxetine and ARA290 reversed CUMS-induced accumulation of Ly6G^hi^ neutrophils and Ly6C^hi^ monocytes in the meninges without significantly affecting cDCs (Figure 4C).

### ARA290 Reduced Chronic Unpredictable Mild Stress- and Chronic Social Defeat Stress-Induced Microglia Activation

**FIGURE 5:**
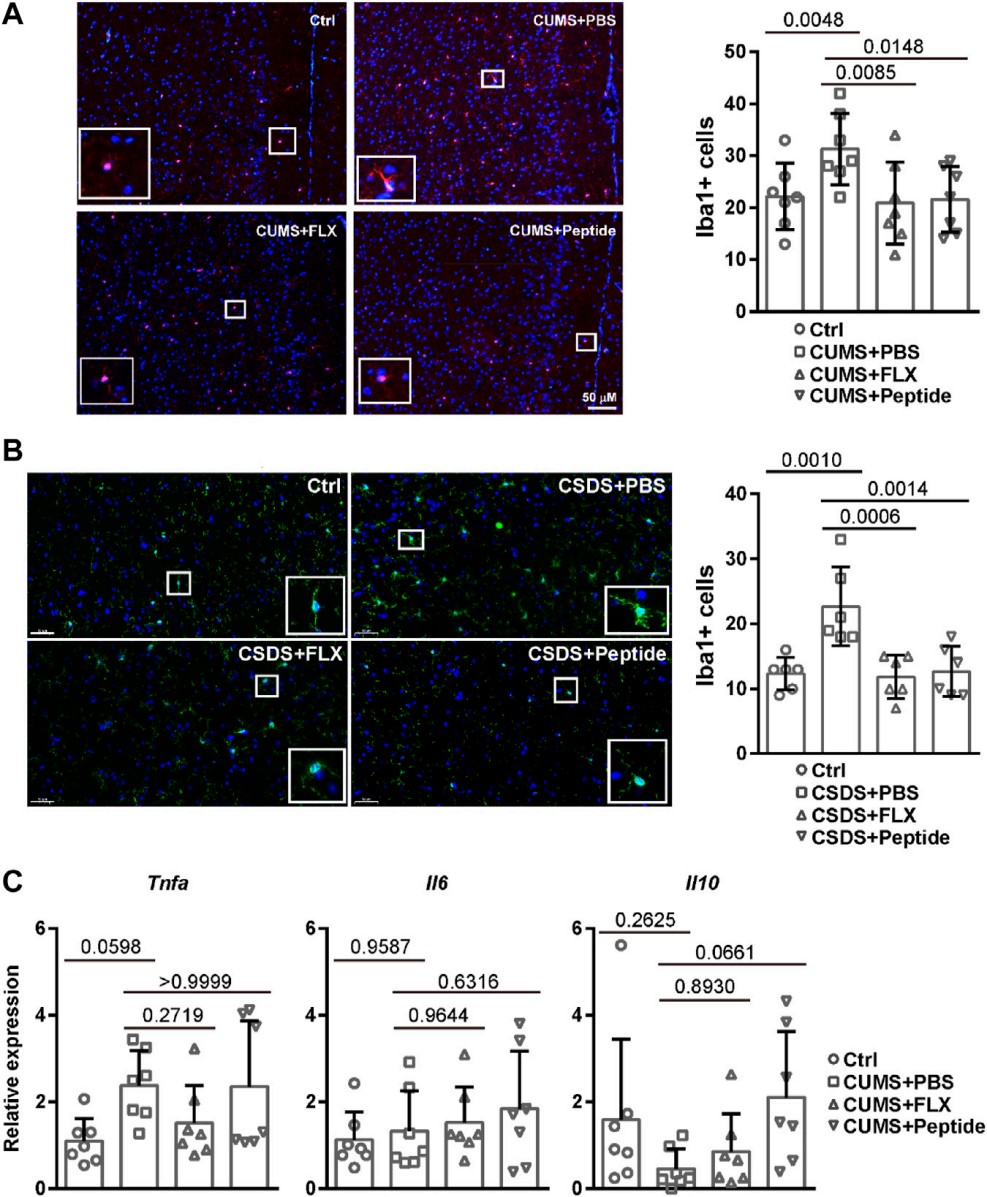
ARA290 reduced CUMS- and CSDS-induced microglia activation: 8-week-old male C57BL/6 mice subjected to the CUMS model (*n* = 7 mice per group) **(A and C)** or 5-week-old male C57BL/6 mice subjected to the CSDS model (*n* = 6 mice per group) **(B)** were treated with fluoxetine (FLX) or ARA290 (peptide) daily. The prefrontal cortex coronal sections were subjected to immunofluorescence staining of Iba1 (scale bar, 50 μM). DAPI was used as a marker for the nucleus **(A and B)**. The mRNA levels of inflammatory cytokines in the prefrontal cortex were determined by quantitative RT-PCR **(C)**. The statistical values between different groups are shown.

Our aforementioned data suggest that the intervention role of fluoxetine and ARA290 is associated with reduced inflammation both in the peripheral and brain borders. Therefore, we set out to measure microglia activation. Microglia activation is associated with Iba1 immunoreactivity (Norden et al., 2016). Immunofluorescence revealed that the CUMS and CSDS depression-like mouse models led to a significantly higher number of Iba1 positive cells in the prefrontal cortex. Both fluoxetine and ARA290 reversed the activation of microglia (Figures 5A and B). We also investigated the expression of inflammatory cytokines in the prefrontal cortex. Quantitative RT-PCR revealed that the CUMS model tended to result in a higher mRNA level of *Tnfa* and fluoxetine showed the tendency to reverse it, although the effects failed to reach statistical significance. By contrast, ARA290 did not affect the mRNA level of *Tnfa* in the CUMS model. There was no difference in the mRNA level of *Il6* between different experimental groups. In addition, the CUMS model tended to lead to a reduced mRNA level of *Il10* and ARA290 treatment showed the tendency to reverse it, although the effects failed to reach statistical significance. By contrast, fluoxetine did not affect the mRNA level of *Il10* in the CUMS model (Figure 5C). These data suggest that fluoxetine and ARA290 inhibit inflammation through different mechanisms.

## Discussion

It has been demonstrated that the anti-inflammatory and cytoprotective effects of EPO are realized through the heterodimeric EPOR/CD131 complex (Brines and Cerami, 2012; Bohr et al., 2013). Therefore, the potential mechanisms of action of ARA290 in preventing the stress-induced depression-like behavior in mice should be attributed to, at least partially, its anti-inflammatory and cytoprotective effects. Even though both fluoxetine and ARA290 inhibited chronic stress-induced peripheral inflammation and microglia activation, the two drugs showed different effects on the expression of inflammatory cytokines, suggesting that the two drugs inhibit inflammation through different mechanisms. It has been reported that fluoxetine inhibits NF-κB activation in microglia/macrophages (Tian et al., 2019) and works as a direct NLRP3 inhibitor to limit inflammasome activation (Ambati et al., 2021). On the other hand, several studies have revealed that EPO induces microglia/macrophage polarization toward M2 whereas such a role was not found for fluoxetine (Wang et al., 2017; Wei et al., 2017; Gu et al., 2021; Wang et al., 2021). EPO activates the JAK2-STAT3 pathway through the heterodimeric EPOR/CD131 complex to promote M2 polarization (Wei et al., 2017; Wang et al., 2021). Therefore, it is reasonable to propose that ARA290 also has such effects. This work is the first to suggest that ARA290 induces microglial polarization toward M2 even though previous studies have demonstrated that ARA290 inhibits microglia/macrophage activation (Liu et al., 2014; Swartjes et al., 2014; Watanabe et al., 2016; Huang et al., 2018).

Our study also indicates that erythropoiesis in the bone marrow was inhibited by chronic stress, which was associated with the tendency of reduced hemoglobin and red cells in the blood. These data are in line with a previous report that CSDS suppressed the level of peripheral blood red cells although the effects on the level of hemoglobin failed to reach statistical significance (Orlovskaya et al., 2018). The underlying mechanism(s) remain elusive. We did not carefully monitor food intake. As both CUMS and CSDS led to reduced body weight, it is possible that mice under chronic stress take less food, which results in partially impaired erythropoiesis in the bone marrow. Another possibility is that CUMS or CSDS triggers chronic inflammation, which inhibits erythropoiesis in the bone marrow (Ganz, 2019). As both fluoxetine and ARA290 inhibited chronic stress-induced systemic inflammation, it is reasonable that the two drugs could reverse or show the tendency to reverse the adverse effects of CUMS and CSDS on erythropoiesis. Because erythroid progenitor cells might gain some immunosuppressive activity (Han et al., 2018; Zhao et al., 2018), it is likely that aberrant erythropoiesis also contributes to the disease progression. Future studies will address these issues.

## Data Availability

The original contributions presented in the study are included in the article/supplementary material, further inquiries can be directed to the corresponding author.
